# Milk Thistle Extract Enhances Lactational Performance in Primiparous Sows: A Comparative Screening of Plant-Derived Additives

**DOI:** 10.3390/ani16193005

**Published:** 2026-09-24

**Authors:** Yuedai Wu, Li Yang, Zongze He, Man Wang, Xiang Zhou, Jian He, Mao Hu

**Affiliations:** 1 Engineering Research Center of Biomass Materials, Ministry of Education, College of Life Sciences and Agri-Forestry, Southwest University of Science and Technology, Mianyang 621010, China; wuyuedai1998@outlook.com (Y.W.); yang1144091526@outlook.com (L.Y.); 15183525782@139.com (M.W.); zhouxiang21wb@163.com (X.Z.); hejiantougao@126.com (J.H.); 2 Institute of Animal Nutrition, Sichuan Agricultural University, Chengdu 611130, China; xxzongze@gmail.com

**Keywords:** milk thistle extract, silymarin, fenugreek, mannan oligosaccharide, primiparous sow, lactation

## Abstract

Primiparous sows frequently fail to meet lactation nutrient demands, but direct comparisons of plant-derived feed additives are scarce. Eighty primiparous Yorkshire sows were assigned to four treatments from 14-day pre-farrowing to 21-day weaning: control (CON), milk thistle extract (MTE, 1% in 100 g/d), fenugreek seed extract (FSE, 1% in 100 g/d), and mannan oligosaccharide (MOS, 1.4% prepartum and 1.8% postpartum in 100 g/d). Production traits were recorded; mechanistic assays were confined to CON and MTE, the group that performed best in the production screen, and are reported as an exploratory, hypothesis-generating comparison. Lactation feed intake differed among groups (*p* = 0.047): MTE sows ate 4.16 kg/d compared to 3.51 kg/d for CON, while FSE (3.73) and MOS (3.47) were intermediate and similar to CON. Only MTE improved litter performance, raising weaning litter weight (+10.6%), piglet weight (+15.4%), and calculated milk yield (+13.1%) versus CON (*p* < 0.05); FSE and MOS showed no significant effects. MTE also increased prolactin (at farrowing and d-14) and catalase (at farrowing) but lowered d-14 cholesterol and HDL. In this two-step trial, MTE was the sole additive enhancing lactational performance, with endocrine and redox changes consistent with improved lactation.

## Simple Summary

Primiparous sows often consume insufficient feed during lactation, which limits milk production and piglet growth. We compared three plant-derived feed supplements, milk thistle extract (MTE), fenugreek seed extract (FSE), and mannan oligosaccharide (MOS), against a control in 80 sows (20 per group). All groups were evaluated for production and reproduction: only MTE increased lactation feed intake relative to the control, whereas backfat and farrowing performance did not differ among groups. Among the three plant additives, only MTE improved piglet growth and calculated milk yield, whereas FSE and MOS showed no such benefit. Detailed blood analyses (prolactin, antioxidant, and metabolic markers) were then performed, as an exploratory follow-up, for the control and MTE groups only, with MTE having been the only additive to improve production traits. MTE-fed sows showed higher prolactin and catalase, together with heavier weaning litters and piglets. The trial was designed as an exploratory, hypothesis-generating comparison of plant-derived additives in primiparous sows.

## 1. Introduction

Modern genetic selection has increased litter size, yet the sow’s capacity to meet the resulting nutritional demands has not kept pace, especially during the periparturient period when requirements peak [1,2]. The gap is most pronounced in primiparous sows, which attain sexual maturity before physical maturity and enter lactation with a limited appetite; reported intakes in first-parity sows frequently fall short of the calculated requirements for maintenance, milk output, and continued maternal growth, as estimated by factorial models of sow energy and protein requirements [3,4,5], leaving little spare capacity before the sow must catabolize body tissue to meet lactation demands [6]. That intake must support the sow’s own growth, milk synthesis, and recovery from parturition [3,4,6,7,8]. First-lactation performance predicts second-parity retention and lifetime productivity [9,10]; consequently, the low intake of primiparous sows may be the mechanistic link connecting early-lactation nutrition with lifetime productivity, given that first-lactation weight loss is associated with reduced embryo survival and weaker second-parity retention [9,10]. Nutritional strategies in this parity should therefore do more than supply nutrients: they should help the sow manage the transition from gestation to lactation. Restrictions on antibiotic growth promoters have redirected interest toward plant-derived feed additives, valued for their antioxidant, anti-inflammatory, and gut-health effects [11].

Milk thistle (Silybum marianum) extract, whose active fraction is silymarin, is a flavonolignan complex with hepatoprotective, antioxidant, and endocrine-modulating activity [11]. Most studies, however, have tested whether a single such additive is effective in isolation; direct, head-to-head comparisons of several plant-derived additives within one herd remain scarce [12], even though producers must eventually choose among them. As a result, the field can report that an extract is effective without establishing which is preferable. The silymarin literature in pigs is instructive because of the contradictions it contains. In gestating gilts, silymarin raised prolactin and lowered oxidative-stress markers without improving mammary development [13,14]; supplied as a non-formulated, high-dose preparation to lactating sows, it produced no clear benefit [15]. However, a micelle-formulated product given from late gestation through lactation improved reproductive performance and serum metabolites at a lower absolute dose [16], and several studies in multiparous sows reported gains in feed intake, milk yield, or redox status [17,18,19,20,21]. Two explanations may account for these differences. First, formulation and bioavailability, rather than absolute dose, can determine efficacy: a low-dose preparation may outperform a high-dose one when delivery differs [15,16]. Second, timing matters. Colostrum composition is largely fixed during the final weeks of gestation, before most supplementation windows open, whereas milk yield during lactation follows the postpartum milieu [22,23,24]; trials that started too late or ended too early may have misread a phase-specific effect as no effect.

Fenugreek seed extract (FSE) and mannan oligosaccharide (MOS) are plausible alternatives, and each carries a rationale that is independent of the hepatoprotective and antioxidant route proposed for silymarin. Fenugreek seed contains steroidal saponins, galactomannan fiber and 4-hydroxyisoleucine, and both the seed and its extracts have a long history of use as galactagogues; in multiparous lactating sows, dietary FSE improved sow and piglet performance and reduced facal *E. coli* counts [25]. MOS is a yeast-cell-wall-derived prebiotic that resists hydrolysis by endogenous enzymes; it binds type-1 fimbriated enterobacteria and modulates gut-associated lymphoid tissue, thereby shifting nutrient partitioning away from immune activation. In sows, MOS shortened the weaning-to-estrus interval and improved piglet growth and immune status [26]. The two additives were therefore included because they plausibly act through galactopoietic and gut-barrier routes distinct from silymarin, so that a null result for either would carry mechanistic information rather than being merely uninformative. Doses followed the ranges reported to be effective in sows [25,26]. In this study, we compared three plant-derived additives, MTE, FSE, and MOS, against a control in primiparous sows. Two parts of the design should be distinguished. The first is the hypothesis, which was formulated in advance and tested in all four groups: we hypothesized that the three additives would not be equivalent in their effects on lactational performance, that MTE would improve lactational performance relative to the control, because the silymarin literature in sows gave a direction to predict, and that the responses to FSE and MOS would be open, because no comparable evidence existed in this parity. The second is the mechanistic stage. Because the study was dimensioned as a screen, mechanistic profiling could not be run on all four groups; it was carried out, as an exploratory follow-up, on the control and on the group that had performed best in the production screen. That choice was made after the production results were available and is therefore post hoc, and the mechanistic comparisons are reported as hypothesis-generating. The two are independent: the a priori hypothesis concerns production performance and stands on its own, whereas the mechanistic stage is a separate, exploratory step nominated by the screen. Samples from all four groups were collected at the outset and were retained.

## 2. Materials and Methods

### 2.1. Animals, Ethics, and Experimental Overview

The animal study protocol was approved by the Institutional Animal Care and Use Committee of Southwest University of Science and Technology (approval no. L2024065) and was conducted in accordance with the guidelines for the ethical treatment of animals. A total of 80 clinically healthy primiparous Yorkshire sows from the same production batch, selected for similar body weight, body condition, and backfat, were enrolled in this trial. Because all animals were primiparous sows, parity was not a confounder. Sows were 240 ± 15 days of age at breeding and weighed 140–170 kg at farrowing. Animals were randomly allocated from the same batch to one of four treatments, CON, MTE, FSE, or MOS, with 20 sows per group (*n* = 20). The four groups were balanced at enrollment with respect to body weight and backfat (*p* > 0.05 for all parameters). Outcome measurements were recorded without blinding to treatment allocation; given the objective, instrument-based nature of the recorded variables, absence of blinding was not expected to introduce bias. Following allocation, sows were housed individually in farrowing crates under commercial farrowing management. Water was available at all times. The experimental period extended from 14 days before the expected farrowing date to weaning at approximately 21 days of lactation (35 days in total). Procedures followed institutional animal-care principles for production-animal research.

### 2.2. Diets, Supplements, and Nutrient Package Preparation

All groups received basal gestation and lactation diets formulated to meet the farm’s standard nutrient specifications for late-gestating and lactating primiparous sows (Table 1). Three commercial plant-derived supplements were used. FSE (fenugreek flavonoids: 12.3%, fenugreek polysaccharides: 9.2%, alkaloids: 1.8%; Shangqiu Tianbang Animal Pharmaceutical Co., Ltd., Shangqiu, China) was provided as a nutrient package containing 1% extract. MTE powder (silymarin content: >80%; Sichuan Hengrui Tongda Biotechnology Co., Ltd., Chengdu, China) was provided as a nutrient package containing 1% MTE; an independent assay of the experimental package confirmed 0.8092 g silymarin per 100 g of package, i.e., approximately 809.2 mg silymarin per sow per day through the 100 g/day top-dress. MOS type I (active content: ≥ 15%; Tangshan Top Biotechnology Co., Ltd., Tangshan, China) was provided as two nutrient packages, containing 1.4% MOS prepartum and 1.8% MOS postpartum. The MTE dose was selected with reference to Wei et al. [18], who reported benefits at 250–500 mg/kg dietary silymarin, and the fenugreek and MOS doses with reference to Hossain et al. [25] (0.1–0.2% FSE) and Duan et al. [26] (400 mg/kg MOS to sows, 800 mg/kg to piglets). To accommodate differences in the feeding level and delivery form across these references and to avoid farm-scale handling issues, each supplement was incorporated into an individual nutrient package rather than blended into the bulk diet. Using standard premix technology for feed additives, rice bran meal was used as the carrier because it is dry, non-caking, chemically stable, and unreactive with the active compounds. Rice bran meal was ground and passed through a 40-mesh sieve, and then mixed with the respective extract to yield the target concentrations. The CON group received 100 g/day rice bran meal; the FSE, MTE, and MOS groups received 100 g/day of their respective nutrient package (FSE and MTE: 1% extract; MOS: 1.4% prepartum and 1.8% postpartum). The packages were measured with a 50 g fixed container and top-dressed twice daily (morning and afternoon), spread evenly over the feed surface. Sows were fed according to the farm routine. During late gestation, a fixed allowance was offered in two meals (08:00 and 17:00), not exceeding 3.5 kg/d. On the day of farrowing, feed was withheld; on postpartum day 2, sows returned to the two-meal gestation allowance, and from postpartum day 4, they had free access to feed offered in four meals per day (08:00, 12:00, 16:00, and 20:00). Water was available at all times. The ingredient composition and nutrient levels of the basal diets are shown in Table 1.

Equal-weight samples of the gestation and lactation basal diets were collected daily, pooled and homogenized to give one composite sample per diet, which was ground to pass a 1 mm screen and stored at −20 °C until analysis. All chemical analyses were performed in duplicate at the Institute of Animal Nutrition, Sichuan Agricultural University (Chengdu, China), following the procedures of AOAC International [27]. Dry matter was determined by oven-drying at 105 °C to constant weight (AOAC 930.15); crude protein (N × 6.25) was determined by the Kjeldahl method on a Kjeltec 8400 analyzer unit (Foss Analytical A/S, Hillerød, Denmark) (AOAC 984.13); crude fat was determined by Soxhlet extraction with petroleum ether on a Soxtec 2050 system (Foss Analytical A/S) (AOAC 920.39); crude ash was determined by incineration at 550 °C in a muffle furnace (AOAC 942.05); calcium was determined by flame atomic absorption spectrophotometry on an AA-6880 spectrophotometer (Shimadzu Corporation, Kyoto, Japan) (AOAC 968.08); and total phosphorus was determined by the vanadomolybdate colorimetric method on a UV-2600 spectrophotometer (Shimadzu) (AOAC 964.06). Amino acids were determined on an L-8900 automatic amino acid analyzer (Hitachi High-Technologies, Tokyo, Japan) after hydrolysis in 6 M HCl at 110 °C for 24 h (AOAC 982.30); sulphur amino acids were oxidized with performic acid before hydrolysis. Crude fibre, total dietary fibre, insoluble dietary fiber, soluble dietary fiber, digestible energy, metabolizable energy, net energy, available phosphorus and digestible amino acids were calculated from the ingredient composition using tabulated nutrient concentrations and standardized digestibility coefficients from NRC (2012) [28].

**Table 1:** Composition and nutrient levels of the basal diets (as-fed basis).

Ingredients ^1^	Gestation Diet	Lactation Diet
Corn	57.66	45.52
Soybean meal, 43% CP	14.40	11.30
Wheat bran	2.20	-
Beet pulp	8.00	2.09
Soybean hull	5.48	0.82
Wheat flour	8.20	25.00
Extruded soybean	-	7.09
Soybean oil	-	1.04
Enzymatically hydrolyzed soybean protein A	-	2.00
L-Lysine HCl, 98.5%	-	0.46
L-Tryptophan, 30%	0.01	0.17
DL-Methionine, 99%	0.18	0.14
Limestone	0.80	1.22
Dicalcium phosphate	1.68	1.38
Salt	0.40	0.40
Magnesium oxide	0.20	0.16
L-Lysine sulfate, 70%	0.16	-
Sodium bicarbonate	0.04	0.42
L-Threonine, 99%	0.06	0.20
L-Valine, 98%	-	0.09
Choline chloride, 60%	0.13	0.10
Premix ^2^	0.40	0.40
Total	100.00	100.00
Nutrient levels ^3^		
Moisture, %	12.64	12.35
Digestible energy, kcal/kg	2924	3323
Metabolizable energy, kcal/kg	2812	3189
Net energy, kcal/kg	2250	2480
Crude protein, %	13.50	16.03
Crude fat, %	2.35	4.66
Crude ash, %	5.10	4.91
Crude fiber, %	5.81	3.20
Calcium, %	0.88	0.90
Total phosphorus, %	0.64	0.60
Available phosphorus, %	0.34	0.30
Total dietary fiber, %	19.52	12.40
Insoluble dietary fiber, %	15.41	10.39
Soluble dietary fiber, %	4.09	2.00
Digestible lysine, %	0.63	1.02
Digestible methionine, %	0.38	0.37
Digestible valine, %	0.52	0.71
Digestible tryptophan, %	0.12	0.20
Digestible threonine, %	0.45	0.66

^1.^ Basal diets were formulated based on National Research Council (2012) standards [28]. ^2.^ The premix provided the following nutrients per kg of diet: vitamin A: 6000 IU, vitamin D_3_: 2100 IU, vitamin E: 46 IU, vitamin K_3_: 2.7 mg, thiamin: 1.0 mg, riboflavin: 5.0 mg, vitamin B_6_: 1.8 mg, vitamin B_12_: 0.02 mg, niacin: 20.0 mg, pantothenic acid: 15.0 mg, biotin: 0.25 mg, folic acid: 5 mg, Mn (as manganese sulfate): 110 mg, Zn (as zinc sulfate): 100.0 mg, Cu (as copper sulfate): 5.40 mg, I (as potassium iodide): 0.30 mg, Se (as sodium selenite): 0.30 mg, Fe (as ferrous sulfate): 100 mg. ^3.^ Nutrient levels: moisture, crude fat, crude ash, crude protein, calcium, and total phosphorus were measured values, whereas the remaining nutrient levels were calculated. Analytical procedures and instrumentation are described in Section 2.2. As-fed basis; ingredient proportions and nutrient levels are expressed as %, except digestible, metabolizable, and net energy, which are kcal/kg.

### 2.3. Productive and Reproductive Measurements

Sow feed intake was summarized for the final 15 days before farrowing and for days 1–21 postpartum. Backfat thickness was measured at the standard P2 position (last rib, 6.5 cm off the midline) at enrollment (approximately 14 days before expected farrowing) and at weaning (day 21), using a V6+ ultrasonic backfat meter (Shenzhen Shengnuowei Technology Co., Ltd., Shenzhen, China); backfat loss was calculated as the difference between the two measurements. Farrowing performance included total born, live-born, healthy-born, weak-born, and stillborn piglets per litter. Litters with fewer than 10 total born piglets were reviewed as potentially non-representative; after verification, none met this criterion, so all groups were retained. Litter birth weight, individual birth weight, weaning litter weight, weaning piglet weight, postnatal death or culling, litter daily gain, and total calculated milk yield from days 1 to 21 were compiled from the farm production records. Litter equalization (cross-fostering) was performed only on the day of birth and on day 3, strictly within treatment group, to standardize litter size following farm routine; no creep feed was provided during lactation. Because cross-fostering was confined to the same treatment group, treatment assignment could not be confounded by fostering.

### 2.4. Colostrum and Serum Sampling

Colostrum samples were collected on the farrowing day before routine litter equalization where feasible. Fat, protein, lactose, urea nitrogen, and dry matter were determined at the Institute of Animal Nutrition, Sichuan Agricultural University (Chengdu, China). Crude protein was determined by the Kjeldahl method on a Kjeltec 8400 analyzer unit (Foss Analytical A/S, Hillerød, Denmark) (AOAC 984.13; N × 6.38); fat was determined by the Röse–Gottlieb gravimetric method (AOAC 905.02); dry matter was determined by oven-drying at 105 °C to constant weight (AOAC 925.10); lactose was determined by the enzymatic UDP-glucose method; and urea nitrogen was determined by the urease–Berthelot method. All determinations were made in duplicate and the mean of the two readings was used. To minimize stress-related variation in endocrine measures, day-14 serum sampling was standardized to a fixed 10:00 AM window after a uniform 1 h feed withdrawal. At farrowing, blood was collected from the sow’s ear vein after delivery of the first piglet (2 × 5 mL vacuum tubes per sow). Samples were allowed to clot at room temperature for 30 min and then centrifuged at 3000 g and 4 °C for 15 min, and the supernatants were aliquoted and stored at −20 °C until assay. The interval between first-piglet delivery and blood collection varied slightly among sows. Serum was obtained from all four treatment groups. In line with the two-step design, detailed endocrinological, hepatic, lipid and antioxidant profiling was confined to the CON and MTE groups. Colostrum was collected and cryopreserved from all four treatment groups at farrowing, so that the material would remain available for future work; in the second, exploratory stage, composition was analyzed in the CON and MTE groups only, and those groups were selected on the basis of their performance in the production screen. The trial was conducted in two stages, which differed in their status. In the first stage, production and reproductive performance were recorded in all four groups, and serum and colostrum were collected from all four groups; this stage tested the a priori hypothesis, and its comparisons were unaffected by the second stage. In the second stage, mechanistic profiling was carried out on the control and on the additive group that performed best in the first stage, which was MTE. The mechanistic stage was not pre-specified: the group was selected after the production results were analyzed, so these comparisons are post hoc and are reported as exploratory. Extending the panel to all four groups would have roughly doubled the number of assays across more than twenty correlated endpoints, which the study was not resourced to support. The endpoints analyzed under this contrast are the colostrum composition, hepatic-function, lipid, hormone, and antioxidant indices reported in Section 3.

Serum alanine aminotransferase (ALT), aspartate aminotransferase (AST), total protein (TP), urea, albumin (ALB), total bile acid (TBA), alkaline phosphatase (ALP), total cholesterol (CHO), high-density lipoprotein cholesterol (HDL), low-density lipoprotein cholesterol (LDL), and triglycerides (TG) were measured on an automatic biochemical analyzer (Model 7020, Hitachi High-Technologies, Tokyo, Japan) using the corresponding commercial kits (Sichuan Maker Bio-technology Inc., Chengdu, China) according to the manufacturer’s instructions. Prolactin (PRL), progesterone (PRG), and cortisol (COR) were determined by porcine-specific enzyme-linked immunosorbent assay kits (Jiangsu Meimian Industrial Co., Ltd., Yancheng, China; catalog nos. MM-0907O1, MM-1205O1 and MM-0686O1, respectively); the intra- and inter-assay coefficients of variation were <10% and <15%, respectively, and the limits of detection were 1.0 ng/mL for PRL, 0.5 ng/mL for PRG, and 1.0 ng/mL for COR. Total superoxide dismutase (T-SOD), glutathione peroxidase (GSH-Px) and catalase (CAT) activities, and malondialdehyde (MDA) and total antioxidant capacity (T-AOC) levels were measured with commercial colorimetric kits (Nanjing Jiancheng Bioengineering Institute, Nanjing, China; catalog nos. A001-1-2, A005-1-2, A007-1-1, A003-1-2, and A015-2-1, respectively) on a microplate reader (Synergy H1, BioTek Instruments, Winooski, VT, USA). All samples were assayed in duplicate, and samples from the two groups were distributed across analytical batches so that group could not be confounded with analytical run.

### 2.5. Calculations

Litter weight gain was calculated as weaning litter weight minus birth litter weight. Calculated milk yield over days 1–21 was then derived as litter weight gain × 4, on the common assumption that approximately 4 kg of sow milk supports 1 kg of litter weight gain; it is reported throughout as a derived estimate and not as a directly measured volume. This litter weight-gain method follows Zhang et al. [16], who likewise calculated milk yield during lactation from piglet growth. Because calculated milk yield is a linear function of litter weight gain, its treatment effect is not independent evidence and is interpreted in the same way as the primary outcome. Alternative direct methods exist (for example, deuterium-oxide dilution [29]), but the litter weight-gain method was chosen because it can be applied routinely in commercial herds.

### 2.6. Statistical Analysis

Data were recorded in Microsoft Excel 2019 and analyzed using IBM SPSS Statistics version 21.0. The experimental unit was the individual sow for feed intake and backfat and the litter for piglet growth and calculated milk yield. Normality of each variable was checked with the Shapiro-Wilk test at α = 0.05 and Q-Q plots; when data were not normally distributed, Welch’s t-test was used, and pooled-variance t-tests were applied to the remaining contrasts. Under a completely randomized design, treatment effects on production and reproductive traits were evaluated by one-way analysis of variance (ANOVA) with Tukey’s test for pairwise comparisons. For mechanistic endpoints, the two groups available (CON and MTE) were compared directly. Results are reported as least-squares means with pooled standard errors of the mean (SEM); mechanistic traits were analyzed using the designated CON-versus-MTE contrast. Significance was declared at *p* < 0.05, and tendencies were noted for 0.05 < *p* ≤ 0.10.

## 3. Results

### 3.1. Sow Feed Intake and Backfat Dynamics

Sow feed intake and backfat dynamics are shown in Table 2. Feed intake during the final 15 days before farrowing did not differ among treatments (*p* = 0.11; Table 2). Numerically, the MOS group ate 12.9% less than the control (2.17 versus 2.49 kg/d), a difference that did not reach significance. From days 1–21 postpartum, feed intake differed among treatments (overall *p* = 0.047; Table 2), with MTE-fed sows consuming more than the control and FSE and MOS showing intermediate values that are not different from the control. Thus, lactation feed intake was increased by MTE, while FSE and MOS showed no clear effect. Backfat did not differ among groups at any stage (all *p* ≥ 0.53; Table 2). The pattern indicates that MTE, which raised intake, did so without evidence of excessive body-tissue mobilization.

### 3.2. Farrowing, Piglet Growth, and Milk Yield

Farrowing performance across the four treatments is shown in Table 3. No trait differed significantly among groups. All comparisons were non-significant (*p* ≥ 0.19; Table 3), and only modest numerical variation was observed. The primary lactational endpoints are reported below. Piglet growth and calculated milk yield were compared across the four treatments (Table 4); together with the production screen, this identified MTE as the additive that most improved lactation. Birth litter weight (*p* = 0.67; Table 3) and birth piglet weight (*p* = 0.56; Table 4) were nearly identical among groups. By weaning, however, litters from MTE-fed sows were heavier than all other groups. Weaning litter weight increased from 65.55 to 72.51 kg in the MTE group, whereas FSE and MOS remained similar to or lower than the control, with MTE exceeding the other three groups (*p* = 0.02; Table 4). Weaning piglet weight showed the same pattern (MTE shows the highest; *p* = 0.04; Table 4), as did litter daily gain (*p* = 0.05; Table 4). Postnatal death or culling remained similar among treatments (*p* = 0.91; Table 4). Calculated milk yield from days 1–21 increased from 199.84 to 225.92 kg/litter in the MTE group (*p* = 0.04), whereas FSE and MOS were similar to or lower than the control (Table 4). Because birth weights were comparable and mortality was not reduced, the heavier weaning weights are most consistent with improved milk supply or milk utilization during lactation.

### 3.3. Weaning-to-Estrus Interval

Post-weaning reproductive records were available for the same cohort (Table 5). The 7-day re-service rate was highest in MTE (100%) and lowest in FSE (85%), with the control (88%) and MOS (90%) falling in between. The weaning-to-estrus interval was shortest in MTE (4.28 d) and longest in FSE (5.83 d), with MOS (5.54 d) and the control (5.69 d) being intermediate (SEM 0.29; *p* = 0.32). The weaning-to-estrus interval did not differ among treatments (*p* = 0.32); the 7-day re-service rate is reported as observed values without statistical testing.

### 3.4. Colostrum Composition

In the second, exploratory stage of the trial, colostrum composition was compared between the control and MTE groups on the day of farrowing (Table 6) and was not significantly altered by MTE supplementation; samples from the FSE and MOS groups were collected and stored but were not analyzed, so no inference was made about colostrum composition in those groups. Fat percentage was numerically higher in the MTE group, but sow-to-sow variation precluded significance (*p* = 0.22; Table 6). Protein, lactose, urea nitrogen, and dry matter were almost identical between treatments or showed only small numerical shifts (all *p* ≥ 0.45, except fat). Consequently, the improvement in later piglet growth could not be attributed to a clear change in the measured gross composition of colostrum at farrowing.

### 3.5. Serum Biochemical, Endocrine, and Antioxidant Status

Serum hepatic-function indices are summarized in Table 7. ALT, AST, TP, urea, ALB, and TBA did not differ between groups at either farrowing or day 14. ALP was similar on the day of farrowing (*p* = 0.56) but was lower in MTE-fed sows by day 14 (*p* = 0.049; Table 7). In the lipid profile (Table 8), no treatment effect was detected on the day of farrowing. On day 14, CHO and HDL were lower in the MTE group than in the control (*p* = 0.045 and *p* = 0.04, respectively; Table 8); LDL and TG were also numerically lower on day 14, but neither difference reached significance. At farrowing, serum prolactin was higher in the MTE group (*p* = 0.02), and it remained higher on day 14 (*p* = 0.04; Table 9). Progesterone did not differ on the day of farrowing and tended to be lower in the MTE group on day 14 (*p* = 0.09; Table 9). Cortisol was numerically lower in the MTE group at both time points, with large between-sow variation and no significant effect (Table 9). T-SOD, GSH-PX, and T-AOC did not differ at either sampling point; CAT activity was higher in the MTE group at farrowing (*p* < 0.001; Table 10), whereas the lower day-14 MDA did not reach significance (*p* = 0.08; Table 10).

## 4. Discussion

In this primiparous cohort, MTE supplementation from late gestation through weaning improved lactational performance: greater voluntary feed intake, heavier litter and individual weaning weights, and higher calculated milk yield (litter weight-gain method), with no significant change in colostrum gross composition or farrowing performance. The pattern is consistent with MTE acting primarily as a lactation-supporting rather than a prolificacy-enhancing additive, with the principal response expressed during the postpartum nursing period rather than at parturition. Voluntary feed intake during lactation is a well-recognized bottleneck in primiparous sows, which must simultaneously support their own growth, milk synthesis, and recovery from parturition [1,2]. Lactation feed intake increased from 3.51 to 4.16 kg/d, a magnitude in line with reports that silymarin supplementation increases average daily and total feed intake in lactating sows [17,18]. Even modest improvements in primiparous intake are of practical relevance under commercial conditions, where they can carry through to subsequent reproductive performance.

Because the intake response underlies every other production difference reported here, its plausibility needs to be examined directly. Two routes are conceivable. Silymarin may act through the liver: The periparturient sow carries a substantial hepatic load, and the hepatoprotective and antioxidant properties of silymarin [11,30,31,32] would be expected to preserve hepatocyte function and thus support the metabolic clearance and gluconeogenic capacity on which voluntary intake partly depends. Alternatively, or additionally, silymarin may reduce the oxidative and inflammatory burden of the periparturient transition; pro-inflammatory cytokines are established anorexigenic signals [33,34], so a blunted inflammatory response would release appetite. The present data cannot distinguish between these routes, and they cannot exclude a simpler account either, because feed intake was measured but never manipulated: without a pair-fed control, we cannot say whether the endocrine and redox differences are independent mediators of the performance response or consequences of eating more. What can be said is that the finding is not isolated: higher average daily feed intake has been reported in silymarin-supplemented sows [17,18], and Wei et al. [18] attributed part of the lactational response to improved antioxidant and metabolic status. The magnitude is also internally consistent: a difference of 0.65 kg/d (3.51 versus 4.16 kg/d, +18.5%) is of the order that would be expected to support the additional milk output implied by the heavier litters [35,36].

Across the four-treatment screen (feed intake, farrowing, piglet growth, and calculated milk yield), MTE was the only additive that improved lactation outcomes, with significant increases in feed intake, weaning litter and piglet weights, litter daily gain, and calculated milk yield, whereas FSE and MOS were intermediate or lower than the control; pre-farrowing intake, backfat mobilization, and farrowing performance did not differ among the four groups. The fact that the effect was confined to postpartum intake and piglet growth, rather than to farrowing or backfat, mirrors the temporal biology discussed below and argues against a generalized growth-promoting or repartitioning effect of these additives. Two parts of the design should be distinguished. The first is the hypothesis, which was tested in the full four-group comparison: MTE would improve lactational performance. That comparison is complete in itself and provides the evidence for the production effect reported here. The second is the mechanistic stage, whose purpose was explanatory rather than confirmatory. MTE was taken forward because it was the only additive that improved production endpoints; the aim was to ask why it worked and in which systems the difference was expressed, not to supply additional proof that it worked. Because the group was nominated by the screen, the mechanistic comparisons are not an independent test of the production effect, and we do not use them as one. They are reported as hypothesis-generating: they cannot establish that the endocrine and redox differences caused the production response rather than accompanied it, and they carry no information about FSE and MOS, for which samples have been retained. The two-step design therefore evaluates three plant additives at the production level while examining MTE mechanistically, without declaring a single “best” additive across all metrics.

The absence of clear production responses to FSE and MOS in the present trial is consistent with the view that additive efficacy in sows is parity-dependent. The benefits reported for FSE [25] and MOS [26] were observed in lactating sows, a population that typically differs from primiparous animals in metabolic load, appetite, and mammary development. A primiparous sow must partition nutrients toward her own continued growth as well as milk synthesis, and her response to a given phytogenic may therefore diverge from that of older parities. Mechanistically, silymarin’s antioxidant and endocrine modulation plausibly targets the oxidative and inflammatory burden of the periparturient transition, a burden that may be especially pronounced and especially limiting in first-lactation animals. FSE and MOS, by contrast, are thought to act on galactopoietic and gut-barrier pathways that may have been less rate-limiting under the present conditions. This interpretation is tempered by the study design: serum endocrinology, lipid, and antioxidant markers were profiled only in the control and MTE groups, so subclinical responses of FSE and MOS could not be assessed, and the trial was powered as a screen rather than to detect the smaller effects these additives might exert. Accordingly, the null result for FSE and MOS should be read as “no evidence of benefit under these conditions” rather than as evidence of inefficacy. Other plant-derived preparations have likewise improved sow reproductive performance and the growth of suckling piglets under different production conditions [37], which reinforces that the present null result concerns the specific additive and parity combination tested rather than phytogenics in general. A second numerical pattern deserves comment because it could otherwise be over-read. Pre-farrowing feed intake was approximately 12.9% lower in MOS sows than in controls (2.17 versus 2.49 kg/d), but this difference was not significant (*p* = 0.11) and was accompanied by no change in backfat loss or farrowing performance. We therefore regard it as variation within a screen dimensioned for production traits rather than as a prebiotic effect on voluntary intake; notably, the direction is opposite to the intake stimulation sometimes attributed to MOS in multiparous sows, and it should not be interpreted without confirmation.

The most parsimonious explanation for the heavier weaning weights is the approximately 18.5% increase in lactation feed intake: more nutrients made available to the mammary gland would, by themselves, support greater milk synthesis and piglet growth [35,36]. The present study did not include a pair-fed control or adjust for feed intake as a covariate; therefore, the extent to which PRL elevation or antioxidant changes represent independent mediators rather than correlates of higher intake cannot be determined. The absence of a significant increase in backfat loss nonetheless suggests that enhanced litter growth was not achieved through disproportionate depletion of maternal adipose reserves [6]; rather, the additional nutrients may have been partitioned more efficiently toward mammary secretion, or the elevated appetite may simply have allowed the mammary gland to express a higher proportion of its productive capacity [35,36].

The lactational improvements were accompanied by two key physiological changes: elevated serum prolactin and partial improvement in selected antioxidant markers. At farrowing, prolactin measured in serum was higher in the MTE group (41.21 versus 22.64 mIU/L; *p* = 0.02); day-14 sow serum prolactin also remained higher (25.95 versus 17.89 mIU/L; *p* = 0.04), consistent with the established role of prolactin in sustaining galactopoiesis. At the cellular level, prolactin binding to its receptor on mammary epithelial cells activates the JAK2/STAT5, thereby driving transcription of milk protein genes including beta-casein and alpha-lactalbumin [7]. The concurrent numerical decrease in progesterone (*p* = 0.09, tendency), alongside elevated prolactin, may have exerted a synergistic effect on lactogenic capacity, consistent with the principle that sustained galactopoietic signaling is more influential than any single acute peak [38]. Serum cortisol was also numerically lower in the MTE group at both time points, with the greatest numerical decline at farrowing (36.25 versus 76.67 ng/mL, approximately 53% lower); although the large between-sow variance prevented significance (*p* = 0.22), this direction accords with reduced periparturient stress accompanying improved feed intake and body-condition preservation, and should be interpreted cautiously. Whether MTE directly modulates prolactin secretion, or whether higher feed intake and reduced stress secondarily raise prolactin, remains unresolved and should be tested with pair-feeding and repeated hormonal sampling. Regarding antioxidant status, catalase activity was selectively elevated in serum at farrowing (34.25 versus 13.94 U/mL; *p* < 0.001) without concurrent changes in superoxide dismutase, glutathione peroxidase, or total antioxidant capacity, suggesting targeted modulation of peroxide-dependent redox pathways rather than nonspecific antioxidant upregulation. Such selectivity is biologically expected, as antioxidant defense is compartmentalized and enzyme-specific, and dietary phytochemicals may modulate one arm of the redox system without uniformly elevating every marker [11,30,31]. Parturition and the onset of lactation constitute a periparturient oxidative challenge in which hydrogen peroxide is a principal reactive species [30]; catalase, whose substrate is H_2_O_2_, is therefore the enzyme most directly positioned to buffer that burst, so its selective elevation at farrowing is consistent with an adaptive rather than a damaging redox response. The liver, with its central role in peroxisomal oxidase activity and high catalase content, is a plausible source of the elevated serum catalase activity, although contributions from mammary or immune-cell redox changes during the periparturient inflammatory transition cannot be excluded [32]. The catalase finding was derived from serum at a single periparturient time point and, although reliable in magnitude (*p* < 0.001), it lacked concordant changes in other redox markers; it should therefore be regarded as hypothesis-generating rather than definitive. In sows, silymarin administration has likewise been reported to raise circulating prolactin [14], supporting a plausible mechanistic link to the higher prolactin observed here.

The absence of treatment effects on colostrum composition and farrowing performance has a plausible temporal explanation. Colostrum synthesis is predominantly shaped by late-gestation immunological and endocrine processes, including immunoglobulin G transport across the mammary epithelium and hormonal shifts in the final week of gestation, which are largely established before the supplementation window began approximately 14 days before farrowing [22,23,24]. When botanical extracts have been supplied from earlier in gestation, colostrum composition and immunoglobulin content have been shown to respond [39], which suggests that the present 14-day pre-farrowing window was short relative to the processes that determine colostrum quality. By contrast, lactation volume during days 1–21 is more heavily influenced by ongoing suckling stimulus, prolactin support, nutrient intake, and mammary blood flow, all of which were altered in favor of the MTE group. This interpretation is supported by the production data: birth litter weight and individual birth weight were essentially identical between groups, whereas by weaning, litter and individual piglet weights and calculated milk yield were markedly higher (Table 4). Previous silymarin studies in sows have yielded seemingly inconsistent results, yet these become more interpretable when biological context is considered: studies in gestating gilts or lactating multiparous sows reported limited benefits [13,15], whereas investigations using different formulations and feeding windows observed favorable lactational responses [12,16,17,19,20,21].

Several practical aspects of the study design warrant comment. Endocrine and redox profiling were assessed at two postpartum time points, and although formal correction for multiple comparisons was not applied across the multiple endpoints evaluated, several associations fell close to the significance threshold (ALP, CHO, HDL, and the milk-yield-derived endpoints); because no correction for multiplicity was applied, these are reported as numerical patterns only. In addition, the farrowing-day catalase increase was substantial in magnitude (*p* < 0.001, pooled SEM: 1.03 U/mL) and is consistent with a periparturient shift in antioxidant status that provides a basis for mechanistic follow-up. Follow-up studies incorporating repeated sampling, direct lactation measurement, and mechanistic endpoints (including mammary transcriptomics, inflammatory cytokine profiling, repeated hormonal sampling, and circulating silymarin quantification) will further delineate the causal pathways underlying these observations. From a practical perspective, MTE supplementation could serve as a phytogenic adjunct in primiparous lactation programs, particularly in herds where low lactation feed intake remains a persistent challenge despite adequate management; factors such as feeding frequency and peripartal feeding strategy have also been shown to influence sow body condition and colostrum yield [40,41]. Future research should prioritize standardized MTE formulations with matched carrier controls, pharmacokinetic validation, and economic evaluation of downstream outcomes including weaning-to-estrus interval and second-parity retention. In the present cohort, the numerically higher 7-day re-service rate (100% versus 88%) and shorter weaning-to-estrus interval (4.28 versus 5.69 d) in MTE sows point to possible downstream benefits, but neither reached significance and both should be read in that light; the within-treatment-group cross-fostering design (days 0 and 3) makes fostering an unlikely confounder of the production responses. The present findings support MTE as a promising nutritional strategy for supporting lactational performance in primiparous sows and provide a rationale for larger-scale investigations to confirm these benefits and optimize delivery protocols.

## 5. Conclusions

Among the additives evaluated, only MTE improved lactational performance in primiparous sows, increasing lactation feed intake, weaning litter and piglet weights, and calculated milk yield relative to the control, whereas FSE and MOS showed no significant effects. These production gains were accompanied by higher serum prolactin in early lactation and by greater catalase activity at farrowing, pointing to endocrine and antioxidant pathways that may contribute to the response. Two features of the design bound this conclusion: Calculated milk yield was derived from litter weight gain rather than measured directly. On the production evidence alone—consistent across feed intake, litter growth, and calculated milk yield—MTE warrants larger confirmatory trials with standardized formulations, direct lactation measurement, feed-intake adjustment, and pharmacokinetic validation. The endocrine and redox findings are exploratory, come from a contrast nominated by the screen rather than pre-specified and are not part of that evidence; they serve only to motivate the follow-up work.

## Figures and Tables

**Table 2:** Effects of treatments (CON, MTE, FSE, and MOS) on sow feed intake and backfat thickness.

Items	Control	MTE	FSE	MOS	SEM	*p* Value
Feed intake, day-15 to farrowing, kg/d	2.49	2.57	2.34	2.17	0.02	0.11
Feed intake, days 1–21 postpartum, kg/d	3.51 ^b^	4.16 ^a^	3.73 ^b^	3.47 ^b^	0.24	0.047
Pre-farrowing backfat, mm	15.68	15.21	15.75	15.26	0.47	0.63
Weaning backfat, mm	13.07	12.47	13.53	12.91	0.48	0.53
Backfat loss to weaning, mm	2.87	3.33	2.91	2.77	0.58	0.70

Values are least-squares means ± pooled SEM (*n* = 20 sows/treatment). ^a,b^ Means within a row without a common superscript differ (*p* < 0.05). Treatment effects were assessed by one-way ANOVA followed by Tukey’s test.

**Table 3:** Effects of treatments (CON, MTE, FSE, and MOS) on farrowing performance of primiparous sows.

Item	Control	MTE	FSE	MOS	SEM	*p* Value
Total born, piglets/litter	12.00	13.08	11.92	12.37	0.28	0.23
Live-born, piglets/litter	11.57	12.67	11.42	12.73	0.27	0.19
Healthy-born, piglets/litter	11.43	12.17	11.42	11.95	0.27	0.19
Weak-born, piglets/litter	0.14	0.50	0.42	0.38	0.08	0.46
Stillborn, piglets/litter	0.43	0.42	0.50	0.46	0.09	0.99
Birth litter weight, kg	15.59	16.03	15.33	15.87	0.44	0.67

**Table 4:** Effects of treatments (CON, MTE, FSE, and MOS) on piglet growth and calculated milk yield.

Item	Control	MTE	FSE	MOS	SEM	*p* Value
Weaning litter weight, kg	65.55 ^b^	72.51 ^a^	64.37 ^b^	63.91 ^b^	2.17	0.02
Birth piglet weight, kg	1.46	1.40	1.38	1.42	0.05	0.56
Weaning piglet weight, kg	5.64 ^b^	6.51 ^a^	5.88 ^b^	5.69 ^b^	0.21	0.04
Litter daily gain, kg/d	2.38 ^b^	2.69 ^a^	2.18 ^b^	2.22 ^b^	0.10	0.05
Postnatal death/culling, piglets/litter	0.64	0.60	0.57	0.61	0.19	0.91
Calculated milk yield ^1^, days 1–21, kg/litter	199.84 ^b^	225.92 ^a^	196.16 ^b^	192.16 ^b^	8.13	0.04

Values are least-squares means ± pooled SEM (*n* = 20 sows/treatment). ^a,b^ Means within a row without a common superscript differ (*p* < 0.05). Treatment effects were assessed by one-way ANOVA followed by Tukey’s test. ^1^ Estimated as (weaning litter weight − birth litter weight) × 4; a derived estimate, not a directly measured volume.

**Table 5:** Effects of treatments (CON, MTE, FSE, and MOS) on post-weaning reproductive outcomes of primiparous sows.

Item	Control	MTE	FSE	MOS	SEM	*p* Value
7-day re-service rate, %	88	100	85	90	-	-
Weaning-to-estrus interval, d	5.69	4.28	5.83	5.54	0.29	0.32

Values for post-weaning outcomes are based on 20 sows per treatment. The weaning-to-estrus interval was analyzed by one-way ANOVA followed by Tukey’s test. Means within a row did not differ (*p* > 0.05); superscript letters are therefore not assigned. The 7-day re-service rate is presented as observed values; SEM and statistical analysis were not performed.

**Table 6:** Effects of MTE versus CON on colostrum composition on the farrowing day.

Item	Control	MTE	SEM	*p* Value
Fat, %	5.21	6.62	0.56	0.22
Protein, %	18.60	18.61	0.66	1.00
Lactose, %	2.86	3.03	0.12	0.50
Urea nitrogen, mg/dL	66.48	67.90	2.45	0.79
Dry matter, %	34.07	35.12	0.66	0.45

**Table 7:** Effects of MTE versus CON on serum hepatic-function indices.

Item	Time	Control	MTE	SEM	*p* Value
ALT, U/L	Farrowing day	38.05	38.92	2.54	0.87
	D14	36.54	38.60	2.35	0.68
AST, U/L	Farrowing day	28.23	30.13	2.58	0.73
	D14	28.90	26.14	2.14	0.55
TP, g/L	Farrowing day	60.42	61.39	0.98	0.64
	D14	64.64	62.85	1.14	0.46
Urea, mmol/L	Farrowing day	4.27	4.17	0.19	0.80
	D14	5.57	5.57	0.16	1.00
ALP, U/L	Farrowing day	43.32	46.09	2.21	0.56
	D14	46.28 ^a^	25.47 ^b^	5.47	0.049
ALB, g/L	Farrowing day	39.09	40.24	0.47	0.24
	D14	38.92	38.59	0.43	0.72
TBA, µmol/L	Farrowing day	25.06	19.43	3.35	0.44
	D14	33.90	29.09	3.80	0.59

ALT—Alanine Aminotransferase; AST—Aspartate Aminotransferase; TP—Total Protein; Urea—Urea (Blood Urea Nitrogen); ALP—Alkaline Phosphatase; ALB—Albumin; TBA—Total Bile Acid. ^a,b^ Means within a row without a common superscript differ (*p* < 0.05).

**Table 8:** Effects of MTE versus CON on serum lipid indices.

Item	Time	Control	MTE	SEM	*p* Value
CHO, mmol/L	Farrowing day	2.00	2.05	0.10	0.81
	D14	2.24	1.91	0.08	0.045
HDL, mmol/L	Farrowing day	0.86	0.89	0.06	0.85
	D14	1.09	0.90	0.05	0.04
LDL, mmol/L	Farrowing day	1.08	1.11	0.06	0.83
	D14	1.06	0.96	0.03	0.12
TG, mmol/L	Farrowing day	0.44	0.31	0.05	0.25
	D14	0.28	0.23	0.02	0.25

CHO—Cholesterol; HDL—High-Density Lipoprotein; LDL—Low-Density Lipoprotein; TG—Triglyceride.

**Table 9:** Effects of MTE versus CON on serum hormones.

Item	Time	Control	MTE	SEM	*p* Value
PRL, mIU/L	Farrowing day	22.64	41.21	4.14	0.02
	Day 14	17.89	25.95	2.01	0.04
PRG, ng/mL	Farrowing day	14.01	12.54	0.78	0.37
	Day 14	15.49	12.61	0.86	0.09
COR, ng/mL	Farrowing day	76.67	36.25	15.50	0.22
	Day 14	69.58	47.49	11.69	0.39

PRL—Prolactin; PRG—Progesterone; COR—Cortisol.

**Table 10:** Effects of MTE versus CON on serum antioxidant indices.

Item	Time	Control	MTE	SEM	*p* Value
T-SOD, U/mL	Farrowing day	106.23	106.76	8.24	0.98
	Day 14	77.32	76.69	5.15	0.96
MDA, µmol/L	Farrowing day	4.62	4.13	0.20	0.23
	Day 14	4.55	3.23	0.38	0.08
GSH-PX, U/mL	Farrowing day	337.14	349.29	8.69	0.52
	Day 14	320.43	335.65	15.57	0.65
CAT, U/mL	Farrowing day	13.94	34.25	1.03	<0.001
	Day 14	23.14	23.45	3.67	0.97
T-AOC, mM	Farrowing day	0.29	0.28	0.01	0.85
	Day 14	0.29	0.30	0.01	0.69

T-SOD—Total Superoxide Dismutase; MDA—Malondialdehyde; GSH-PX—Glutathione Peroxidase; CAT—Catalase; T-AOC—Total Antioxidant Capacity.

## Data Availability

The raw data supporting the conclusions of this article will be made available by the authors on request.
